# Trends in Pain Medication Initiation Among Patients With Newly Diagnosed Diabetic Peripheral Neuropathy, 2014-2018

**DOI:** 10.1001/jamanetworkopen.2020.35632

**Published:** 2021-01-28

**Authors:** Jungwei Fan, Molly Moore Jeffery, W. Michael Hooten, Nilay D. Shah, Rozalina G. McCoy

**Affiliations:** 1 Division of Digital Health Sciences, Department of Health Sciences Research, Mayo Clinic, Rochester, Minnesota; 2 Mayo Clinic Robert D. and Patricia E. Kern Center for the Science of Health Care Delivery, Mayo Clinic, Rochester, Minnesota; 3 Division of Health Care Policy and Research, Department of Health Sciences Research, Mayo Clinic, Rochester, Minnesota; 4 Department of Anesthesiology and Perioperative Medicine, Mayo Clinic School of Medicine, Rochester, Minnesota; 5 Division of Community Internal Medicine, Department of Medicine, Mayo Clinic, Rochester, Minnesota

## Abstract

This cohort study assesses trends in the rates of initiation of pain medication among patients with newly diagnosed diabetic peripheral neuropathy and the types of pain medication prescribed from 2014 to 2018.

## Introduction

Diabetic peripheral neuropathy (DPN) affects approximately half of people living with diabetes.^1^ Approximately half of patients with DPN have pain^2^ resulting in debility, disability, and impaired quality of life.^3^ Clinical guidelines recommend use of anticonvulsants, antidepressants (serotonin-norepinephrine reuptake inhibitors, tricyclic antidepressants), or topical analgesics for painful DPN owing to their demonstrated efficacy and safety in this context.^2,4^ To promote safe and evidence-based pain management, we examined initiation of pain medication among adults with newly diagnosed DPN.

## Methods

This retrospective cohort study used electronic health record data from the Mayo Clinics in Rochester, Minnesota; Phoenix, Arizona; and Jacksonville, Florida, health systems. We examined first-line analgesic medications prescribed to adults with a new diagnosis of DPN between January 1, 2014, and December 31, 2018. This study was approved by the Mayo Clinic institutional review board. Informed consent was waived owing to the use of preexisting population-level electronic health record data. The study followed the Strengthening the Reporting of Observational Studies in Epidemiology (STROBE) reporting guideline.

Included medications were prescribed during the first year after diagnosis and had not been prescribed in the preceding 12 months (nonopioids) or 3 months (opioids). Medications were regarded as concurrent if they were prescribed within 2 weeks of each other and were categorized as (1) guideline recommended (pregabalin, gabapentin, and serotonin-norepinephrine reuptake inhibitors); (2) opioids, which are not recommended; and (3) acceptable (topical analgesics, tricyclic antidepressants, and other anticonvulsants) (eTable 1 in the Supplement).^2,4^

New diagnosis of DPN was established using *International Classification of Diseases, Ninth Revision* and *International Statistical Classification of Diseases and Related Health Problems, Tenth Revision* codes preceded by a 2-year period without these diagnoses. Logistic regression models examined demographic and clinical (eTable 2 in the Supplement) factors associated with prescriptions for any treatment among all patients and for opioids or guideline-recommended medications specifically among patients who received treatment. To ensure that patients were active in our health systems, we required that any prescription be issued during the 18 months before DPN diagnosis and between 1 month and 1 year after diagnosis. Analyses were performed using R, version 3.6.1 (R Project for Statistical Computing).

## Results

We identified 3495 adults with newly diagnosed DPN, of whom 1406 (40.2%) started a new pain medication after diagnosis (Table). Between 2014 and 2018, the adjusted odds of starting a new pain medication decreased by 35%, from 45.6% (287 of 630) of the cohort in 2014 to 35.2% (243 of 691) in 2018. The odds of treatment initiation were greatest among patients with depression (odds ratio [OR], 1.61; 95% CI, 1.35-1.93), arthritis (OR, 1.21; 95% CI, 1.02-1.43), and back pain (OR, 1.34; 95% CI, 1.16-1.55) and decreased significantly over time among all patients. Among 1406 patients who received treatment, opioids were prescribed to 616 (43.8%), recommended medications to 603 (42.9%), and acceptable medications to 289 (20.6%). Male sex was associated with greater odds of opioid use compared with use of other medications, and presence of fibromyalgia was associated with lower odds of opioid use. Comorbid arthritis was associated with decreased odds of recommended medication use. Crude rates of opioid use decreased over time, whereas rates of recommended medication increased (Figure). However, trends were not significant after adjustment for demographic and clinical factors (Table).

**Table.** Characteristics of Patients With Newly Diagnosed DPN and Factors Associated With Initiation of Pain Medication After DPN Diagnosis

Characteristic	Population, No. (%) (N = 3495)	Odds ratio (95% CI)			
		Any treatment^a^	Use of recommended medications^b^	Use of opioids^b^
Patient age, y				
18-44	173 (4.9)	1 [Reference]	1 [Reference]	1 [Reference]
45-64	1229 (35.2)	1.13 (0.81-1.59)	0.94 (0.56-1.60)	0.95 (0.57-1.63)
65-74	1117 (32.0)	1.02 (0.73-1.45)	0.95 (0.55-1.64)	1.02 (0.60-1.77)
≥75	976 (27.9)	0.86 (0.60-1.24)	1.08 (0.61-1.90)	0.74 (0.42-1.32)
Sex				
Female	1392 (39.8)	1 [Reference]	1 [Reference]	1 [Reference]
Male	2103 (60.2)	0.88 (0.76-1.02)	0.84 (0.67-1.06)	1.26 (1.01-1.59)^c^
Race/ethnicity				
White	3139 (89.8)	1 [Reference]	1 [Reference]	1 [Reference]
Non-White^d^	356 (10.2)	1.17 (0.93-1.47)	1.32 (0.93-1.88)	0.80 (0.56-1.15)
Diabetes medication				
Noninsulin only	1105 (31.6)	1 [Reference]	1 [Reference]	1 [Reference]
Insulin with or without noninsulin	1685 (48.2)	0.95 (0.80-1.12)	0.85 (0.65-1.10)	1.04 (0.80-1.35)
None	705 (20.2)	1.02 (0.84-1.25)	0.82 (0.60-1.12)	1.25 (0.91-1.70)
Diabetes-related complications				
Retinopathy	799 (22.9)	0.93 (0.78-1.10)	0.90 (0.69-1.18)	1.10 (0.84-1.43)
Nephropathy	1300 (37.2)	1.12 (0.96-1.30)	0.83 (0.65-1.05)	1.07 (0.85-1.36)
Cardiovascular disease	1836 (52.5)	1.06 (0.91-1.24)	0.86 (0.67-1.10)	1.18 (0.92-1.51)
Cerebrovascular disease	417 (11.9)	1.13 (0.91-1.40)	0.83 (0.59-1.17)	1.24 (0.89-1.73)
Peripheral vascular disease	796 (22.8)	1.11 (0.93-1.32)	0.88 (0.67-1.16)	1.19 (0.91-1.55)
Other comorbid conditions				
Depression	858 (24.5)	1.61 (1.35-1.93)^c^	1.21 (0.93-1.58)	1.00 (0.76-1.30)
Anxiety	510 (14.6)	0.88 (0.71-1.10)	1.12 (0.81-1.55)	0.88 (0.64-1.22)
Arthritis	923 (26.4)	1.21 (1.02-1.43)^c^	0.76 (0.59-0.99)^c^	1.02 (0.79-1.31)
Back pain	1567 (44.8)	1.34 (1.16-1.55)^c^	0.85 (0.68-1.07)	1.18 (0.94-1.48)
Fibromyalgia	264 (7.6)	1.03 (0.79-1.34)	1.29 (0.87-1.91)	0.67 (0.44-0.99)^c^
Seizure disorder	61 (1.7)	1.11 (0.66-1.88)	0.63 (0.27-1.38)	1.48 (0.69-3.24)
Complex regional pain syndrome	175 (5.0	1.01 (0.74-1.39)	0.75 (0.45-1.23)	1.42 (0.88-2.29)
Postherpetic neuralgia^e^	13 (0.4)	NA	NA	NA
Year of DPN diagnosis				
2014	630 (18.0)	1 [Reference]	1 [Reference]	1 [Reference]]
2015	648 (18.5)	0.87 (0.69-1.09)	1.19 (0.84-1.68)	0.94 (0.67-1.32)
2016	811 (23.2)	0.79 (0.63-0.98)^c^	1.16 (0.83-1.63)	0.78 (0.56-1.08)
2017	715 (20.5)	0.82 (0.65-1.02)	1.21 (0.85-1.72)	0.71 (0.50-1.01)
2018	691 (19.8)	0.65 (0.52-0.82)^c^	1.25 (0.86-1.80)	0.71 (0.49-1.02)

Abbreviations: DPN, diabetic peripheral neuropathy; NA, not applicable.

^a^ Odds of starting any class of analgesic medications were calculated for the whole study cohort of 3495 patients with newly diagnosed DPN.

^b^ Odds of starting individual categories of analgesic medications were calculated for the subset of 1406 patients who started any drug.

^c^ Statistically significant at *P* < .05.

^d^ The non-White race category includes missing and unknown data. Race was ascertained from electronic health record demographic data and was examined owing to potential disparities in pain management between White and non-White individuals.

^e^ Postherpetic neuralgia was not included in the logistic regression analyses owing to small sample size.

**Figure.**
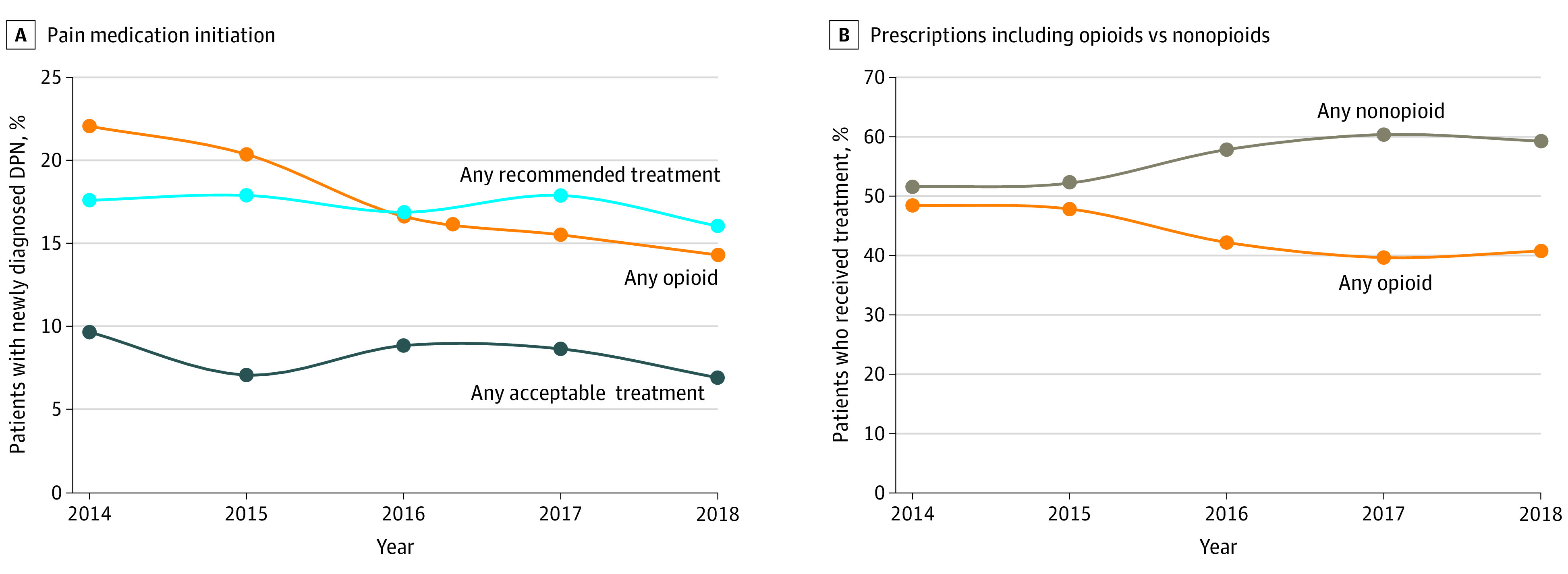
Trends in Pain Medication Initiation Among Patients With Diabetic Peripheral Neuropathy (DPN) A, Patients treated with 2 categories of medications at the same time contribute to both regimens. The denominator was all patients (N = 3495), including those who did not start any treatment. B, Temporal trends in prescriptions for opioids (alone or in combination with other medications) compared with other medications only (recommended, acceptable, or both) among patients who received treatment.

## Discussion

Between 2014 and 2018, initiation of pain medication among patients with newly diagnosed DPN decreased by 35%. Overall, 43.8% patients initiating therapy were prescribed opioids compared with 42.9% who were prescribed guideline-recommended medications. Such high rates of opioid use by patients with DPN, a lifelong pain syndrome, are concerning because safer effective treatment options are available.

Rates of opioid use exceeded those reported previously^5^ likely because we did not exclude patients with preexisting pain or mood disorders, which are common in this population, making our study findings more generalizable. Our analyses are limited by the inability to examine the clinical context of pain management, including pain severity and other potential indications for opioid therapy, or contraindications to guideline-recommended treatments. Our sample size may be underpowered to ascertain factors associated with the choice of specific treatment regimens. Our data could not capture the duration of opioid therapy; thus, some prescriptions may have been for short-term rather than long-term use. In addition, the patients included in the study received care in 3 institutions and, as such, may not be representative of all patients with DPN. Further research is needed to examine DPN management trends nationally, identify factors associated with opioid use and barriers to evidence-based alternatives, and develop interventions to improve DPN management in clinical practice.

## Notes

Supplement. **eTable 1.** Classification of included pain medications **eTable 2.** Comorbidity code sets
