# Supplementary material for: Trends in Pain Medication Initiation Among Patients With Newly Diagnosed Diabetic Peripheral Neuropathy, 2014-2018
Source: JAMA Netw Open. 2021 Jan 28;4(1):e2035632. doi: 10.1001/jamanetworkopen.2020.35632 (PMC7844595; doi:10.1001/jamanetworkopen.2020.35632)
Supplement: Supplement. — eTable 1. Classification of included pain medications eTable 2. Comorbidity code sets [file jamanetwopen-e2035632-s001.pdf]

Supplemental Online Content

Fan J, Jeffery MM, Hooten WM, Shah ND, McCoy RG. Trends in pain medication initiation among patients with newly diagnosed diabetic peripheral neuropathy, 2014-2018. *JAMA Netw Open.* 2021;4(1):e2035632. doi:10.1001/jamanetworkopen.2020.35632

eTable 1. Classification of included pain medications

eTable 2. Comorbidity code sets

This supplemental material has been provided by the authors to give readers additional information about their work.

© 2021 Fan J et al. *JAMA Network Open.*

eTable 1. Classification of included pain medications.

Category	Included Medications
Recommended	Gabapentin
	Pregabalin
	Serotonin-norepinephrine reuptake inhibitors (SNRI): duloxetine, venlafaxine, desvenlafaxine, and desvenlafaxine succinate
Acceptable	*Tricyclic antidepressants (TCA)*: amitriptyline, clomipramine, doxepin, imipramine, trimipramine, desipramine, nortriptyline, protriptyline
	Other anti-convulsants: valproic acid and carbamazepine
	Topical analgesics: lidocaine, capsaicin, isosorbide dinitrate
Opioids	Codeine, dihydrocodeine, hydrocodone, meperidine, oxycodone, propoxyphene, hydromorphone, morphine, buprenorphine, butorphanol, fentanyl, methadone, oxymorphone, tapentadol, and tramadol

© 2021 Fan J et al. *JAMA Network Open.*

eTable 2. Comorbidity code sets. ICD diagnosis codes used to identify chronic health conditions present at the time of diabetic peripheral neuropathy (DPN), applied to the 24 months period preceding the DPN date.

	**ICD-9**	**ICD-10**
Diabetic peripheral neuropathy	249.6x, 250.6x, 357.2	E10.4x, E10.610, E11.4x, E11.610, E13.4x, E13.610
Retinopathy [a]	362.01, 362.03, 362.04, 362.05, 362.06, 362.07, 362.53, 362.81, 362.82, 362.83, 362.02, 379.23, 250.5x, 249.5x, 362.1x, 361.x, 369.x	H35.9x, E08.3x, E09.3x, E10.3x, E11.3x, E13.3x, H35.0x, H35.35x, H35.6x, H35.8x, H33.x, H54.x, H43.1x
Nephropathy [a]	593.9, 586, 250.4x, 249.4x, 580.x, 581.x, 582.x, 583.x, 585.x	E08.21, E08.22, E08.29, E09.21, E09.22, E09.29, E10.21, E10.22, E10.29, E11.21, E11.22, E11.29, E13.21, E13.22, E13.29, N19, N00.x, N03.x, N04.x, N05.x, N18.x
Cardiovascular disease [a]	429.2, 412, 427.5, 427.1, 411.x, 413.x, 414.x, 410.x, 427.3x, 427.4x, 428.x, 440.x, 441.x	I24.x, I20.x, I25.x, I70.x, I21.x, I22.x, I23.x, I48.x, I46.x, I47.x, I49.x, I50.x, I71.x
Cerebrovascular disease [a]	431, 436, 435.x, 433.x, 434.x	I67.81, I61.x, I63.x, I65.x, I66.x, G45.x
Peripheral vascular disease [a]	442.3, 440.21, 443.81, 443.9, 892.1, 040.0, 444.22, 785.4, 250.7x, 249.7, 707.1x	E08.51, E09.51, E10.51, E11.51, E13.51, E08.59, E09.59, E10.59, E11.59, E13.59, E08.621, E09.621, E10.621, E11.621, E13.621, I72.4, I73.89, I73.9, A48.0, I74.3, I96, E08.52, E09.52, E10.52, E11.52, E13.52, I70.21x, S91.3x, L97.x
Depression	311, 290.13, 290.21, 290.43, 296.2x, 296.3x, 296.82, 298.0, 301.12, 309.0, 309.1, 309.28	F01.51, F32.xx (except F32.81), F33.xx, F34.8x, F43.21, F43.23
Anxiety	300.0x, 300.2x, 309.2x	F40.x, F41.x, F43.22
Arthritis	711.2x, 714.xx (except 714.81), 715.xx, 720.x, 725	M05.xxx, M06.xxx, M12.0xx, M35.2, M45.x, M46.1, M46.8x, M46.9x, M49.8x, M35.3
Seizure disorder	345.xx	G40.xxx
Post-herpetic neuralgia	053.11, 053.12, 053.13, 053.19	B02.2x
Fibromyalgia	729.1	M79.7
Complex regional pain syndrome	337.2x, 354.4, 355.7x, 355.9	G90.5x
Back pain	721.xx, 722.xx 723.xx, 724.xx	M45.x, M46.x, M47.x, M48.x, M49.x, M50.x, M51.x, M53.x, M54.x

[a] Diabetes complications were ascertained using the Diabetes Complications Severity Index.[1]

REFERENCES

1. Chang HY, Weiner JP, Richards TM, Bleich SN, Segal JB. Validating the adapted Diabetes Complications Severity Index in claims data. *Am J Managed Care.* 2012;18(11):721-726.

© 2021 Fan J et al. *JAMA Network Open.*
